# Yu Ping Feng San, an Ancient Chinese Herbal Decoction Containing Astragali Radix, Atractylodis Macrocephalae Rhizoma and Saposhnikoviae Radix, Regulates the Release of Cytokines in Murine Macrophages

**DOI:** 10.1371/journal.pone.0078622

**Published:** 2013-11-11

**Authors:** Crystal Y. Q. Du, Roy C. Y. Choi, Ken Y. Z. Zheng, Tina T. X. Dong, David T. W. Lau, Karl W. K. Tsim

**Affiliations:** 1 Division of Life Science and Center for Chinese Medicine, The Hong Kong University of Science and Technology, Hong Kong, China; 2 Department of Biology, Hanshan Normal University, Chaozhou, Guangdong, China; Istituto Superiore di Sanità, Italy

## Abstract

Yu Ping Feng San (YPFS), a Chinese herbal decoction, is composed of Astragali Radix (AR; Huangqi), Atractylodis Macrocephalae Rhizoma (AMR; Baizhu) and Saposhnikoviae Radix (SR; Fangfeng) in a weight ratio of 1∶2∶1. Clinically, YPFS has been widely used to regulate immune functions; however, the action mechanism of it is not known. Here, we addressed this issue by providing detail analyses of chemical and biological properties of YPFS. By using rapid resolution liquid chromatography coupled with mass spectrometry, fifteen chemicals deriving from different herbs of YPFS were determined, and which served as a control for the standardization of the herbal extract of YPFS. In general, the amounts of chosen chemical markers were higher in a preparation of YPFS as compared to that of single herb or two-herb compositions. In order to reveal the immune functions of YPFS, the standardized extract was applied onto cultured murine macrophages. The treatment of YPFS stimulated the mRNA and protein expressions of pro-inflammatory cytokines via activation of NF-κB by enhancing IκBα degradation. In contrast, the application of YPFS suppressed the expressions of pro-inflammatory cytokines significantly in the lipopolysaccharide (LPS)-induced chronic inflammation model. In addition, YPFS could up regulate the phagocytic activity in cultured macrophages. These results therefore supported the bi-directional immune-modulatory roles of YPFS in regulating the releases of cytokines from macrophages.

## Introduction

Compatibility in traditional Chinese medicine (TCM) is a very specialized methodology having a combination of different herbs as to form a formulated herbal decoction (named as Fu Fang) [1], [2]. Unfortunately, the complexity of chemical composition avoids the delineation of molecular mechanisms of a herbal decoction. Yu Ping Feng San (YPFS) composes of Astragali Radix (AR; Huangqi; the root of *Astragalus membranaceus* (Fisch.) Bunge or *Astragalus membranaceus* (Fisch.) Bunge var. *mongholicus* (Bunge) P.K. Hsiao), Atractylodis Macrocephalae Rhizoma (AMR; Baizhu; the rhizomes of *Atractylodes macrocephala* Koidz.) and Saposhnikoviae Radix (SR; Fangfeng; the roots of *Saposhnikovia divaricata* (Turcz.) Schischk.) in a weight ratio of 1∶2∶1. YPFS was described in Dan Xi Xin Fa by Zhu Danxi in Yuan Dynasty (A.D. 1279–1368) of China. Literally, the name YPFS means Jade Screen, i.e. forming a solid screen to protect our body. AR, called “the senior of all herbs” in the Essentials of the Materia Medica (A.D. 1694), is considered the best immune tonic herb in stabilizing and strengthening the protective “Qi” [3]–[7]. AMR is bitter, sweet and warm in nature, and which is beneficial for anti-inflammation, anti-hepatotoxic, anti-ulcer, anti-obesity, anti-cancer and so on [8]–[11]. SR is used to expel “wind”, to relieve exterior syndrome, to eliminate dampness, to relieve convulsion and diarrhea [12]–[14]. Having these herbs, YPFS is being used to replenish “*Qi*” and to consolidate the superficies and arrest perspiration, i.e. clinical symptoms having spontaneous sweating, frequent colds, aversion to wind, fatigue and a pale and shiny complexion. Clinically, YPFS has been applied in anti-viral or anti-bacterial effects, which indeed has been shown to prevent viral infections including severe acute respiratory syndrome (SARS) and enhance cellular immunity [15]–[18]. In addition, panaxynol was revealed in YPFS as an active ingredient by using splenocyte binding-HPLC analysis [19]. However, the chemical composition and biological efficacy of YPFS are still rather limited.

Inflammatory response and phagocytosis are the important host defense mechanisms in innate immune system providing rapid and nonspecific immune protections. Clinically, YPFS was indicated to prevent colds and to treat upper respiratory tract infection. Therefore, the biological effects of YPFS were firstly illustrated by inflammatory response and phagocytic activity in cultured macrophages. Nuclear factor kappa-light-chain-enhancer of activated B cells (NF-κB) is an ubiquitous transcription factor that governs the expressions of a wide array of pro-inflammatory cytokines [20]. Interleukin 1β (IL-1β), interleukin 6 (IL-6) and tumor necrosis factor α (TNFα) are the key pro-inflammatory cytokines playing critical roles in the process of inflammation. Activation of NF-κB could prevent the attacks from the invaders via initiation of many downstream immune response genes, e.g. IL-1β, IL-6 and TNFα, whereas a loss of control leads to pathological conditions, such as severe inflammation, auto-immune disease, and inflammation-associated oncogenesis [21]. In parallel, phagocytosis by macrophages is critical for the uptake and degradation of infectious agents and senescent cells [22].

In order to demonstrate the immune-modulatory function of YPFS, we firstly established chemical criteria for a standardized YPFS before various immunological assays. In cultured macrophages, application of YPFS was able to induce the inflammatory response by determining mRNA and protein expressions of pro-inflammatory cytokines, NF-κB transcriptional activity and degradation of NF-κB inhibitory protein IκBα as well as phagocytic activity. Moreover, the role of YPFS in anti-inflammation effects was revealed by determining the mRNA and protein expressions of pro-inflammatory cytokines in the lipopolysaccharide (LPS)-induced chronic inflammation model.

## Results

### Preparation of standardized YPFS

**Figure 1:**
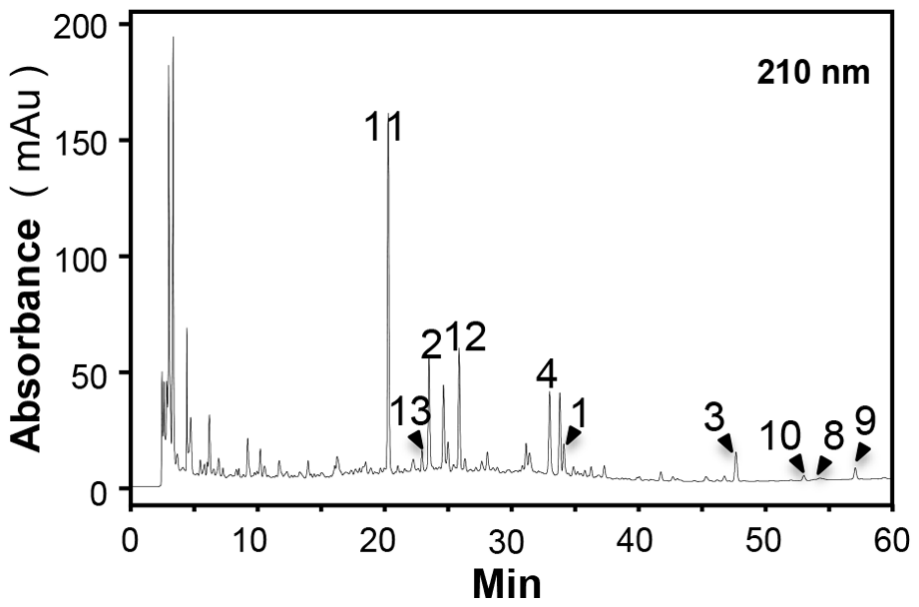
Typical HPLC fingerprint of YPFS at 210 nm. Forty mg/mL YPFS was subjected to HPLC analysis, and the chemical fingerprint was revealed by a DAD detector. Prim-O-glucosylcimifugin (11), scopoletin (13), calycosin-7-O-β-D-glucoside (2), 5-O-methylvisammioside (12), ononin (4), calycosin (1) formononetin (3), atractylenolide III (10), atractylenolide I (8) and Atractylenolide II (9) were identified in the HPLC fingerprint of 210 nm. The chemical markers were numbered according to **Fig. S1**. Representative chromatograms are shown, *n* = 3.

According to the ancient preparation method of YPFS, AR, AMR and SR were boiled together under moderate heating in water, and indeed this was the method being used commonly today in clinical applications. The final extractive was about 52.13±2.46% for YPFS, 33.99±2.25% for AR extract, 67.82±0.38% for AMR extract, and 37.22±0.88% for SR extract (*n* = 3). In order to standardize YPFS chemically, a typical HPLC fingerprint of YPFS at absorbance of 210 nm was developed (**Figure 1**): the fingerprint was used to ensure the detection of the chosen chemical markers from the herbal extracts. More important this fingerprint served as an index for identification of YPFS. As a first step to standardize the extract of YPFS, we developed a rapid LC-MS method to simultaneously identify different chemicals from the three herbs, as a means of quality assessment. In this case, the quantity of these chemicals might be used not only for quality control of YPFS, but also for the elucidation of the compatible principle. Fifteen chemical markers were selected based on their abundant amounts in the herbs and their immune-modulatory effects, as reported in the literatures. These chemicals included: (i) AR-derived flavonoids: calycosin-7-O-β-D-glucoside, calycosin, ononin and formononetin; (ii) AR-derived saponins: astragaloside IV, III and II; (iii) AMR-derived sesquiterpenoids: atractylenolide I, II and III; (iv) SR-derived chromones prim-O-glucosylcimifugin and 5-O-methylvisammioside; and (v) SR-derived coumarins: scopoletin, isopsoralen and psoralen [23]–[26].

**Figure 2:**
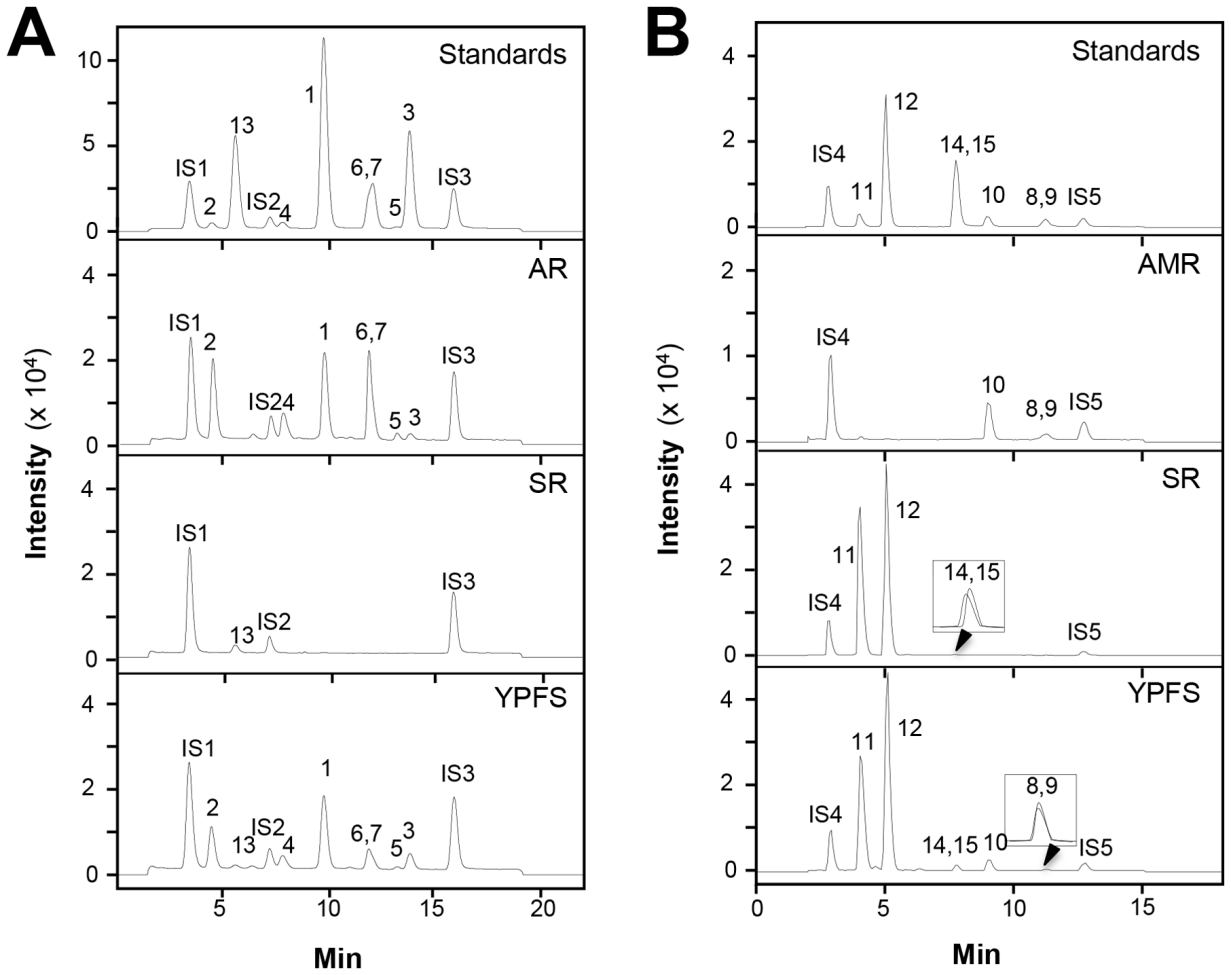
Typical RRLC-DAD-QQQ MS/MS chromatograms of 15 chemical markers in YPFS. (**A**): The identification of calycosin-7-O-β-D-glucoside (2), scopoletin (13), ononin (4), calycosin (1), astragaloside III (6), astragaloside IV (7), astragaloside II (5), formononetin (3), aesculetin (IS1), Rg1 (IS2) and chrysin (IS3) was made by a MS detector in the negative mode. Representative chromatograms are shown, *n* = 3; (**B**): The identification of prim-O-glucosylcimifugin (11), 5-O-methylvisammioside (12), psoralen (14), isopsoralen (15), atractylenolide III (10), atractylenolide II (9), atractylenolide I (8), esculin (IS4), crytotanshinone (IS5) was made by a MS detector in the positive mode. The chemical markers were numbered according to **Fig. S1**. Representative chromatograms are shown, *n* = 3.

The chemical structures of fifteen chosen chemical markers were shown **in Figure S1**. To reveal the fifteen chemicals within a LC-MS analysis, both positive and negative modes were used here (**Fig. 2A and B**). These results were to establish the chemical criteria for quality assurance of YPFS. The fragmentor voltage and collision energy values were optimized to obtain the highest value of the chemicals including the internal standards (**Table S1 and Table S2**). To validate the analytic method, the linearity, sensitivity, precision, repeatability and accuracy of the analytes were determined. For the linearity, the calibration curve of each chemical was constructed using a range of concentrations of working standards, and each line was based on six different concentrations (**Table S3**). The LOD and LOQ were used to evaluate the sensitivity. The LOD was estimated with a signal 3 times higher than that of the baseline noise while the LOQ was 10 times higher. The assay precision was determined by intra-day and inter-day variations, which were performed by analyzing standard solutions during a single day (*n* = 6) and on 3 executive days (*n* = 6), respectively. For repeatability test, five independent samples were prepared. The accuracy was evaluated as the percentage recovery of analytes in the spiked samples. The recoveries were calculated by the following formula: Recovery (%)  = 100× (amount found-original amount)/amount spiked. RSD was used to describe precision, repeatability and recovery (**Table S4**). These results were verified in determining the amount of fifteen chemicals with YPFS.

Having the aforementioned method in standardizing YPFS, a standardized YPFS was recommended to contain no less than the amounts of (i) AR-derived flavonoids: calycosin-7-O-β-D-glucoside, calycosin, ononin and formononetin; (ii) AR-derived saponins: astragaloside IV, III and II; (iii) AMR-derived sesquiterpenoids: atractylenolide I, II and III; (iv) SR-derived chromones prim-O-glucosylcimifugin and 5-O-methylvisammioside; and (v) SR-derived coumarins: scopoletin, isopsoralen and psoralen (**Table 1**). In addition, the water extracts of various herbs, including AR, or AMR, or SR, were also standardized as listed in **Table 1**.

**Table 1:** Quantitative assessment of fifteen chemicals in YPFS, AR, AMR and SR extracts.

Marker chemical	YPFS (mg/g)	AR (mg/g)	AMR (mg/g)	SR (mg/g)
**Calycosin**	0.229±0.001	0.859±0.009	-	-
**Calycosin-7-O-β-D-glucoside**	0.443±0.004	1.709±0.021	-	-
**Formononetin**	0.256±0.006	1.174±0.021	-	-
**Ononin**	0.621±0.003	2.185±0.024	-	-
**Astragaloside II**	0.073±0.027	0.332±0.106	-	-
**Astragaloside III**	0.108±0.008	0.356±0.021	-	-
**Astragaloside IV**	0.111±0.002	0.344±0.009	-	-
**Atractylenolide I**	0.034±0.002	-	0.035±0.003	-
**Atractylenolide II**	0.044±0.001	-	0.057±0.004	-
**Atractylenolide III**	0.309±0.006	-	0.320±0.012	-
**Prim-O-glucosylcimifugin**	0.695±0.013	-	-	3.021±0.019
**5-O-methylvisammioside**	0.469±0.014	-	-	1.824±0.027
**Scopoletin**	0.005±0.000	-	-	0.046±0.003
**Psoralen**	0.005±0.000	-	-	0.021±0.003
**Isopsoralen**	0.005±0.000	-	-	0.024±0.003

One mg/mL YPFS, or other herbal extract, was subjected to LC-MS to quantified the amount of the selected 15 chemical markers. Values are expressed in mg/g from dried extracts of YPFS, AR, AMR and SR, Mean ± SD, where *n* = 3, each with triplicate samples.

### Herb-to-herb interaction in chemical solubility

**Figure 3:**
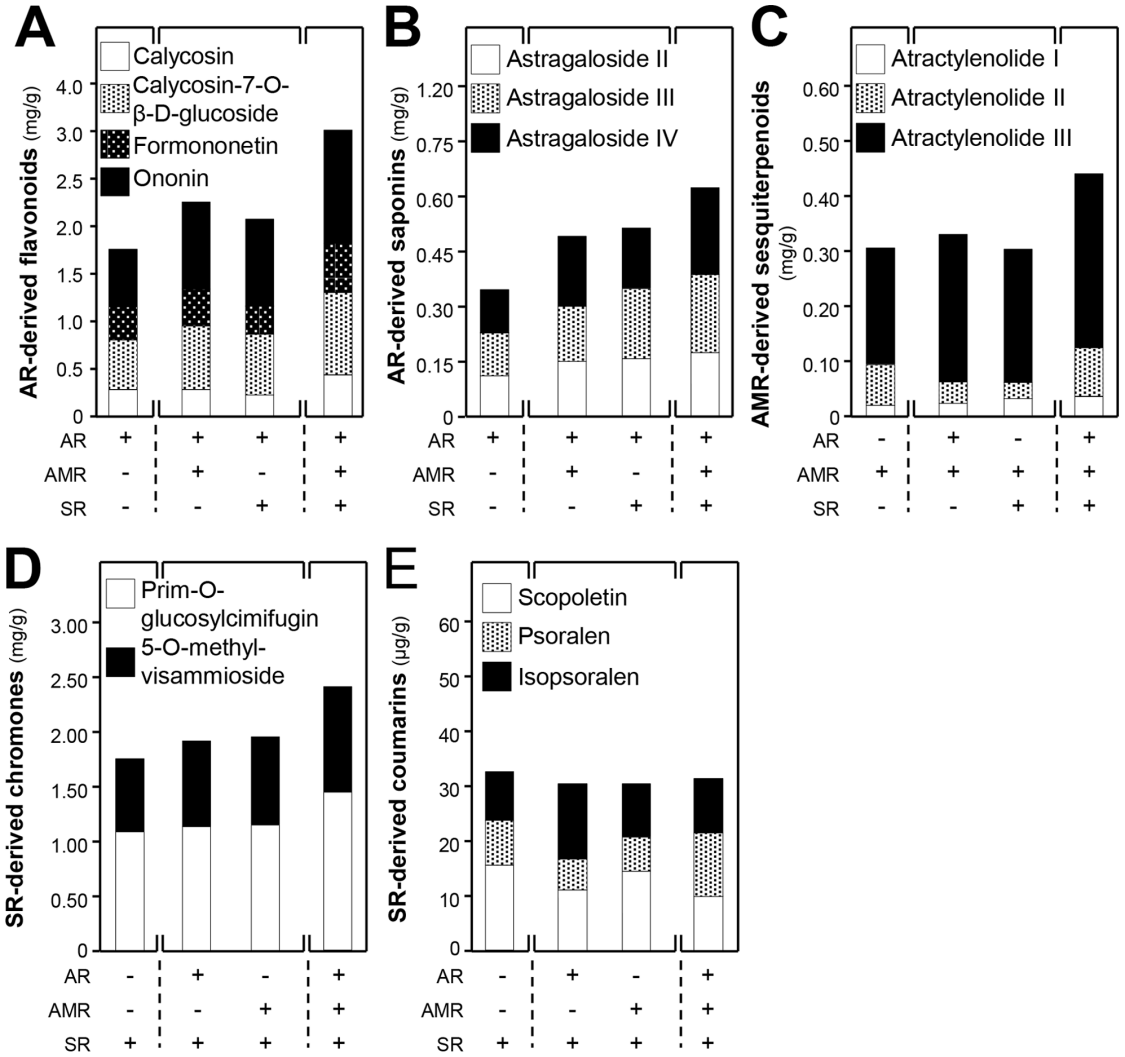
The solubilities of chemical markers in YPFS. The chemical markers including AR-derived flavonoids (**A**) and saponins (**B**), AMR-derived sesquiterpenoids (**C**), SR-derived chromones (**D**) and coumarins (**E**) were determined in water extracts of different herbs, a combination of two herbs, and YPFS. Values are expressed in mg/g or µg/g of dried herb, where *n* = 3.

The solubilities of chemicals during boiling in different combination of herbs are rather complicated, and which subsequently may affect the extraction efficiency of different chemicals as well as its possible biological functions [23], [27]–[30]. In order to reveal the herb-to-herb interaction in chemical solubilities, the amounts of chemicals, extracted in the form of single herb, two herbs together and three herbs together (i.e. YPFS extract), were measured. Different compositions of the herbs, based on the herb ratio in YPFS, were boiled together under standardized protocol as to test the variation of chemical solubility. The solubilities of AR-derived flavonoids, including calycosin-7-O-β-D-glucoside, calycosin, ononin and formononetin, were much better in YPFS (**Fig. 3A**). The increase solublities could also be revealed when AR was boiled together with either AMR or SR. The AR-derived saponins, including astragaloside II, III and IV, showed similar increase in YPFS when AR was boiling with AMR and SR together (**Fig. 3B**). Again, the increase solublities were revealed in boiling together with AMR or SR. The AMR-derived sesquiterpenoids, including atractylenolide I, II and III, showed robust increase (at least by 30% increase) in boiling as YPFS (**Fig. 3C**). The SR-derived chromones, including prim-O-glucosylcimifugin and 5-O-methylvisammioside were increased in a preparation of YPFS (**Fig. 3D**). In contrast, the amount of SR-derived coumarins, including scopoletin, isopsoralen and psoralen were slightly decreased when SR was boiled as in YPFS (**Fig. 3E**). The decrease was similar in a case of boiling together with AR or AMR. Thus, the amounts of those chosen chemical markers were higher, in general, in a preparation of YPFS as compared to that of a single herb, which suggested that the herbal mixture indeed could provide better chemical composition. In addition, the chemical analysis could serve a parameter for chemical standardization and repeatability of the decoction preparation. These values served the minimal amount of chemicals for a standardized YPFS being prepared for each time, and which could be used as a quality index for the biological studies.

### YPFS induces the expression of pro-inflammatory cytokines

**Figure 4:**
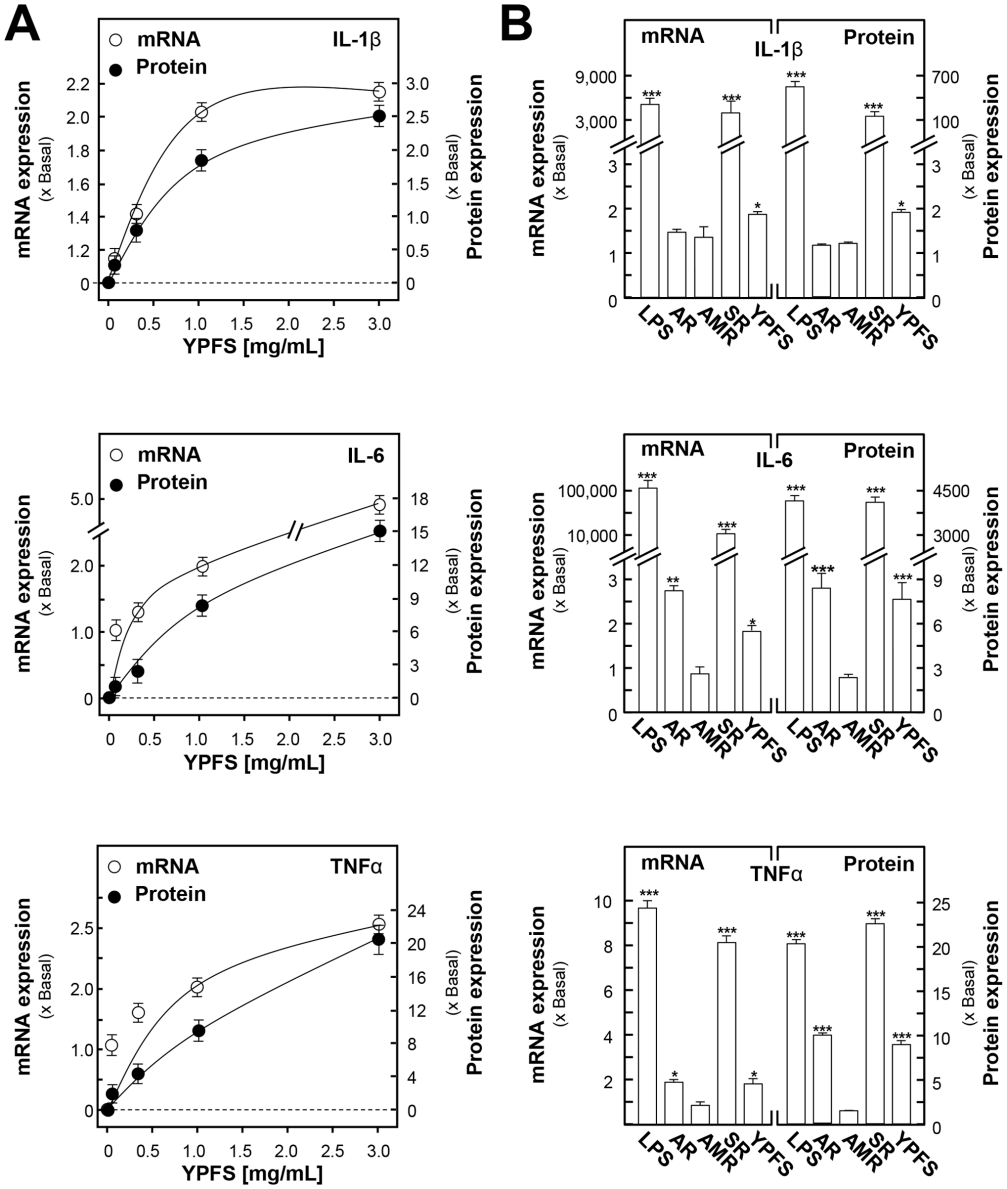
YPFS induces the expression of pro-inflammatory cytokines in cultured macrophages. Cultured macrophages were treated with the herbal extracts for 24-inflammatory cytokines (IL-1 β, IL-6 and TNFα), respectively. The expression levels of pro-inflammatory cytokines were revealed by real time PCR and multiplexed bead-based immunoassay. GAPDH was used as an internal control for normalization in RT-PCR. The protein expression level was normalized to total protein content of each cell culture well. LPS (1 µg/mL) served as a positive control. (**A**): The mRNA and protein expressions of IL-1β, IL-6 and TNFα mRNAs under the treatments of various dosages of YPFS, up to 3 mg/mL. (**B**): The mRNA and protein expressions of IL-1β, IL-6 and TNFα under the treatments of AR, AMR, SR and YPFS extracts, all at 1 mg/mL. Values are expressed as the fold of increase to basal reading (untreated cultures), and in Mean ± SD, where *n* = 4, each with triplicate samples. *p<0.05; * * p<0.01.

Before screening for immune-modulatory functions of YPFS, 3-(4,5-dimethylthiazol-2-yl)-2,5-diphenyltetrazolium bromide (MTT) assay was carried out to detect the cytotoxicity of all the herbal extracts in cultured macrophages. Serial concentrations of herbal extracts (i.e. 0.03, 0.1, 0.3, 1, 3 mg/mL) were applied onto cultured macrophages for 24 hours, and followed by the MTT cell viability assay. The results indicated that all the tested herbal extracts exhibited low toxicity to macrophages, and the maximum dose could be at 3 mg/mL in the following experiments (**Fig. S2**). In order to reveal the immune-modulatory functions of YPFS at molecular level, NF-κB pathway and its downstream genes (e.g. pro-inflammatory cytokines) were our targets of investigation. Cultured macrophages were treated with different YPFS, or AR, or AMR, or SR herbal extracts for 24 hours, and then the transcript and protein expressions of pro-inflammatory cytokines were determined. Under the treatment of YPFS in cultured macrophages, the mRNA and protein expressions of IL-1β, IL-6 and TNFα were stimulated by YPFS in a dose-dependent manner (**Fig. 4A**). The maximal induction was achieved at about 2 to 20 folds under the treatment of 1–3 mg/mL of YPFS in all cases (**Fig. 4A**). The best induction was under the treatment of SR extract: this extract stimulated the mRNA expressions of cytokines by 8- to 10,000-fold and protein expressions by 20- to 4,000-fold (**Fig. 4B**). A robust induction was observed in the expression of IL-6 under the effect of SR extract (**Fig. 4B**). The extracts of AR could also induce the cytokine expressions, and the effect in general was similar to that of YPFS treatment. Here, LPS served as a positive control.

**Figure 5:**
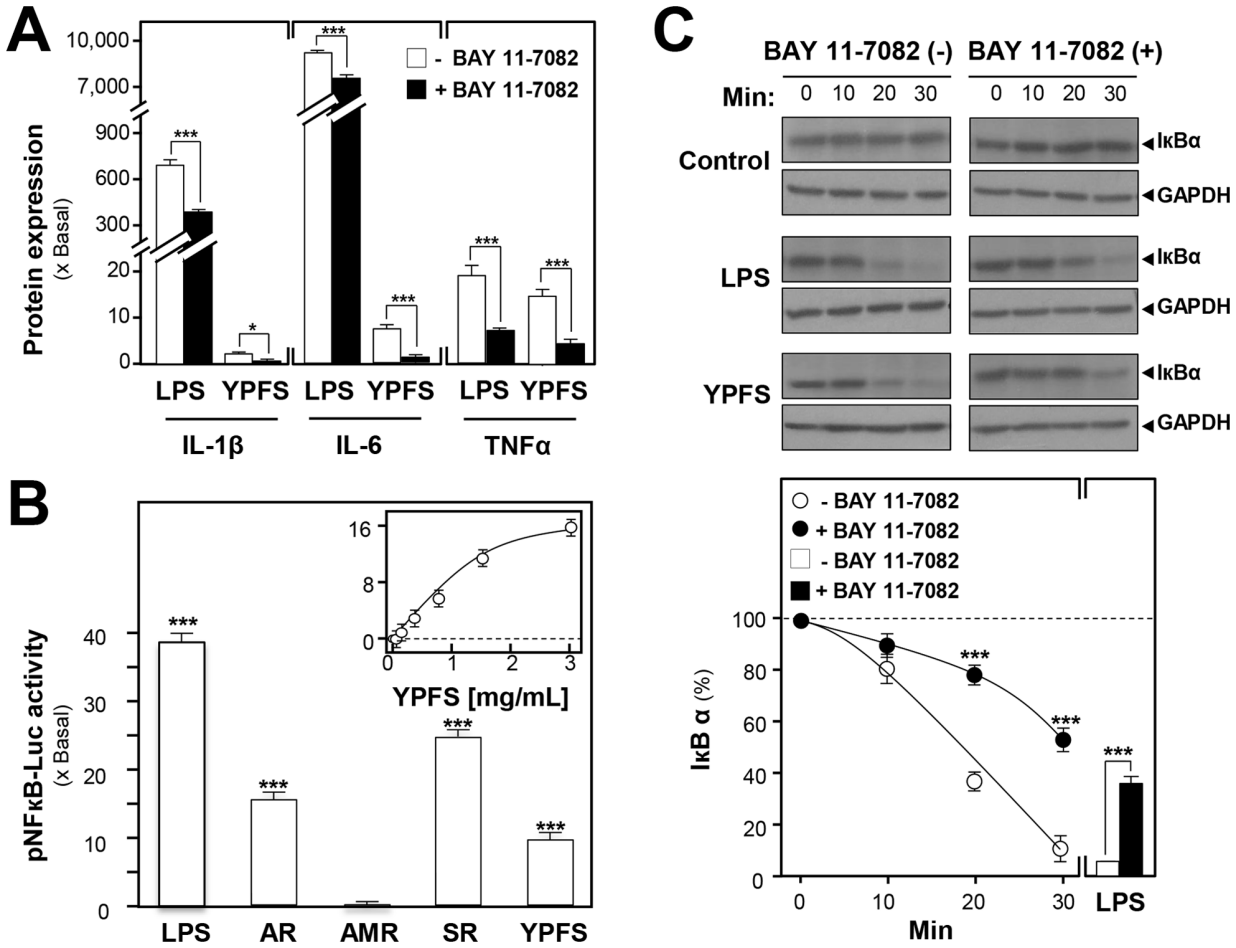
YPFS induces expression of pro-inflammatory cytokines via activation of NF-κB by enhancing the degradation of IκBα in cultured macrophages. (**A**): Effects of BAY 11-7082 on protein expressions of pro-inflammatory cytokines inducing by YPFS in cultured macrophages. Macrophages were treated with herbal extracts for 24 hours in the present or absent of a specific NF-κB inhibitor BAY 11-7082 (5 µM). The cultured medium was collected to analyze the protein expressions of pro-inflammatory cytokines using multiplexed bead-based immunoassay. (**B**): The induction of pNF-κB-Luc by the extracts of AR, AMR, SR and YPFS in cultured macrophages. A luciferase-reporter containing five NF-κBs and a down-stream luciferase reporter gene, namely as pNF-κB-Luc, was used in the transfected macrophages. Cultured macrophages, transfected with pNF-κB-Luc, were treated with the herbal extracts for 24 hours. LPS (1 µg/mL) was used as a positive control. The cell lysates were subjected to luciferase assay to measure the transcriptional activity driven by NF-κB. (**C**): Effects of BAY 11-7082 on YPFS-induced degradation of IκBα in cultured macrophages. Cultures were incubated with BAY 11-7082 for 3 hours prior to stimulation with 3 mg/mL YPFS for the indicated times. Cell lysates were analyzed by western blot using indicated antibody for IκBα and GAPDH. Values are expressed as the fold of increase to basal reading (untreated cultures), and in Mean ± SD, where *n* = 4, each with triplicate samples.

NF-κB is well-known to play a pivotal role in regulating the expressions of cytokines and chemokines in acute phase inflammatory response. To clarify the activation effects of YPFS on expression of pro-inflammatory cytokines, a specific NF-κB inhibitor BAY 11-7082 was utilized for this purpose. Pre-treatment with 5 µM BAY 11-7082 to cultured macrophages for 3 hours, the protein expressions of pro-inflammatory cytokines, stimulated by YPFS, could be significantly suppressed about 2- to 2,000-fold (**Fig. 5A**). To identify the potential NF-κB activators from YPFS, a luciferase-reporter construct having the repeats of NF-κB responsive elements (i.e. pNF-κB-Luc) was employed here. In pNF-κB-Luc transfected cells, the herbal extracts were applied. The promoter-driven luciferase activity was induced by application of YPFS in the transfected macrophages: the induction was in a dose-dependent manner (**Fig. 5B**). The maximal induction could be at over 15-fold. In addition, the activation effects of the extracts of AR, AMR and SR were also determined here. Both AR and SR extracts showed a robust activation of pNF-κB-Luc activity, and the SR-induced activation was at ∼25-fold (**Fig. 5B**). The control LPS treatment showed an indication of ∼40-fold. For the mechanistic aspect, the effect of YPFS on the degradation of NF- κB inhibitory protein IκBα was determined in the present or absent of BAY 11-7082. After 30 min incubation with LPS, about 90% of IκBα was degraded as compared with the original IκBα. Pre-treatment with 5 µM BAY 11-7082 for 3 hours, LPS could only induce 60% degradation of IκBα. More importantly, 3 mg/mL of YPFS could induce the degradation of IκBα in a time-dependent manner (0, 10, 20 and 30 min). By application of Bay 11-7082 for 3 hours, the effects of YPFS in degrading IκBα could be partially blocked (**Fig C**). All of the results consistently support the idea that YPFS induces the expressions of pro-inflammatory cytokines via activation of NF-κB through enhancing degradation of IκBα.

### YPFS stimulates phagocytic activity

**Figure 6:**
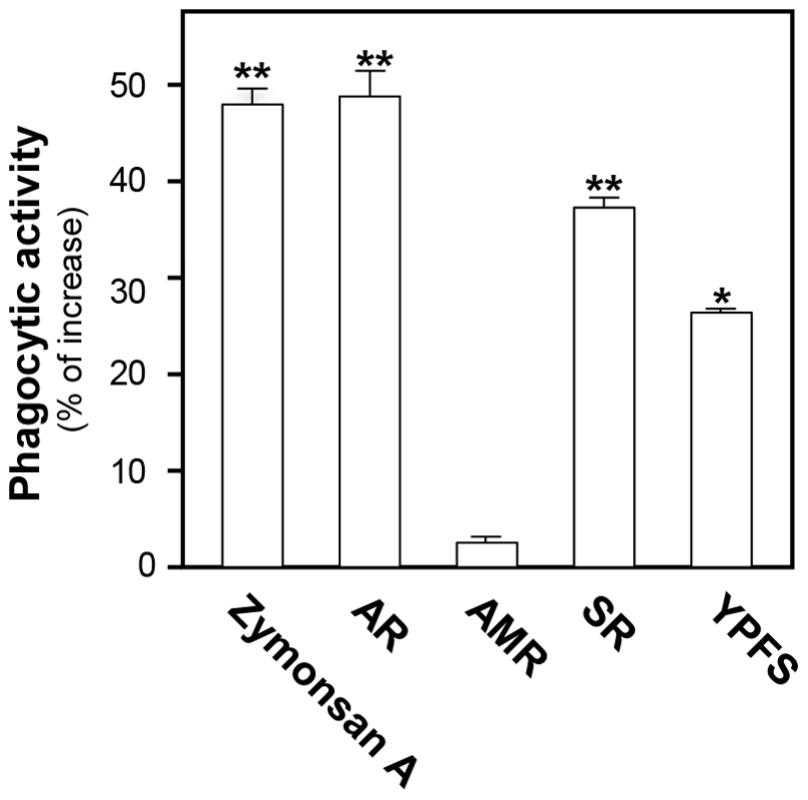
YPFS induces the phagocytic activity in cultured macrophages. Cultured macrophages were pretreated with 1/mL extract of AR, AMR, SR and YPFS separately for 3 hours; thirty µg/mL Zymonsan A was used as a positive control. The procedures of phagocytosis were followed by the protocol of Vybrant TM phagocytosis assay kit (V-6694). Values were expressed as the fold of stimulation to basal reading, and in Mean ± SD, where *n* = 4, each with triplicate samples. *p<0.05; **p<0.01.

Phagocytosis of pathogens by macrophages initiates the innate immune response, which in turn orchestrates the adaptive response. Zymosan A, a carbohydrate complex derived from yeast cell walls and an essential reagent in the study of phagocytosis activation, was used as a positive control. Here, we tested the effects of YPFS and extracts from various herbs on the phagocytic activity of cultured macrophages. Macrophages were pre-incubated with different herbal extracts, and then treated with fluorescein-labeled *E. coli* K-12 bio-particles. The macrophages engulfed more particles by the application of AR, SR and YPFS, indicating that they could increase phagocytic activity as compared with control group (**Fig. 6**).

### YPFS possesses anti-inflammation effects

**Figure 7:**
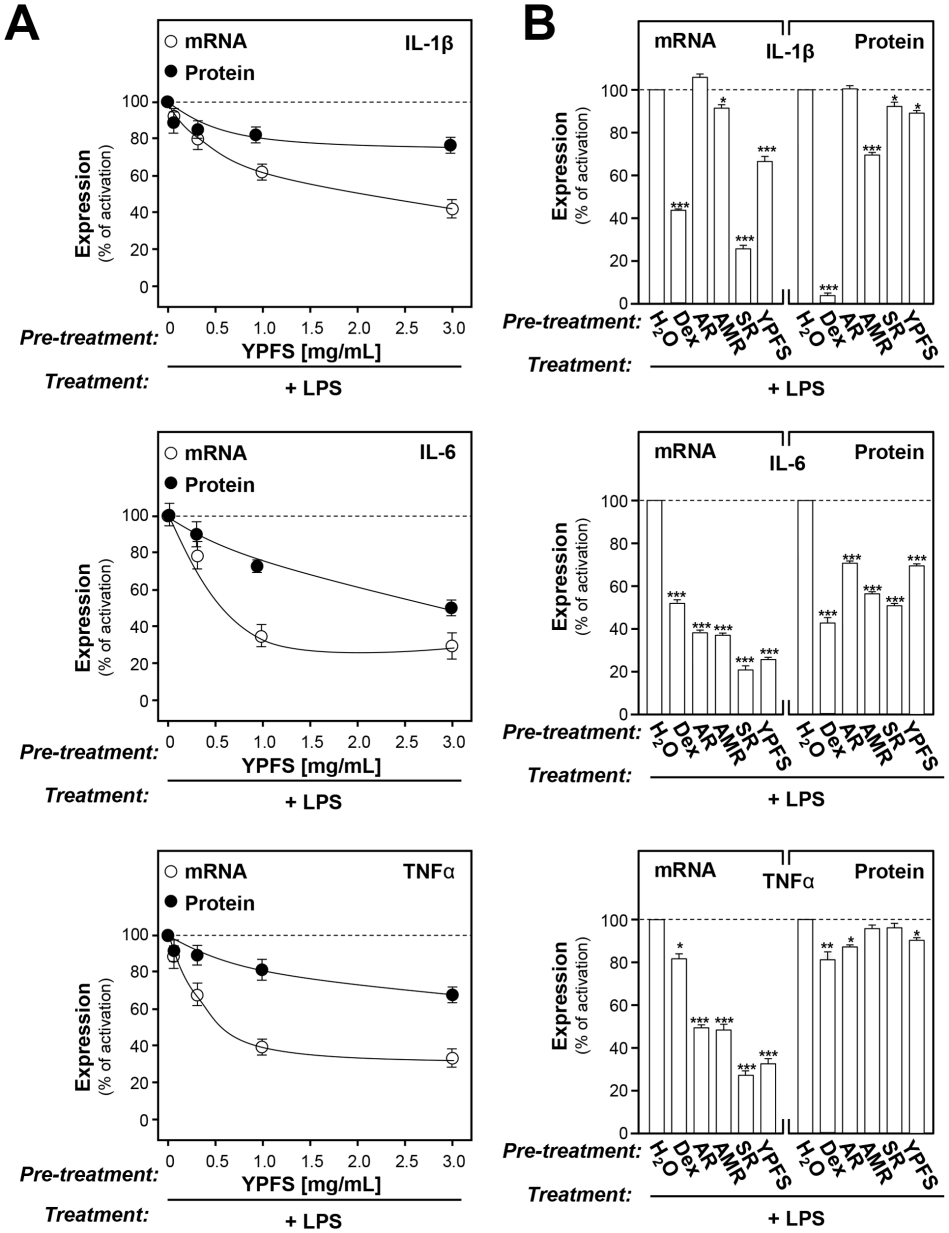
YPFS suppresses the LPS-induced expression of pro-inflammatory cytokines in cultured macrophages. Cultured macrophages were pretreated with 1/mL herbal extracts for 3 hours. Then, LPS (1 µg/mL for gene expression, 0.5 µg/mL for protein expression) was applied onto the cultures for 24 hours to mimic the chronic inflammation. The levels of mRNAs encoding pro-inflammatory cytokines IL-1β, IL-6 and TNFα were revealed by real time PCR, while GAPDH was served as an internal control for normalization. The protein expressions of pro-inflammatory cytokines were revealed by multiplexed bead-based immunoassay. Here, 10 µM Dex served as a positive control. Values were normalized to total protein content of each cultured well. Values are expressed as % of the LPS-induced activation, and in Mean ± SD, where *n* = 3, each with triplicate samples. *p<0.05; ***p<0.001.

Here, we would like to further explore the anti-inflammation effect of YPFS. Specifically, we investigated the effects of YPFS in suppressing the expressions of pro-inflammatory cytokines in the LPS-induced chronic inflammation model. One µg/mL or 0.5 µg/mL of LPS was added to macrophages to mimic the chronic inflammation for determination of gene and protein expressions of pro-inflammatory cytokines, respectively. This treatment was a well-known model for studying anti-inflammation [31]. Here, YPFS and other herbal extracts were applied onto cultured macrophages in testing the inhibition effects on the LPS-induced expression of pro-inflammatory cytokines. In LPS-induced cytokine expressions, YPFS suppressed the transcript and protein expressions of pro-inflammatory cytokines in a dose-dependent manner (**Fig. 7A**). YPFS suppressed IL-1β protein expression by ∼20%, IL-6 protein expression by ∼40%, and TNFα protein expression by ∼25% in the LPS-induced chronic inflammation model (**Fig. 7A**). The extracts from single herb showed the suppression effects as that of YPFS (**Fig. 7B**). The suppression effects of AMR extract were stronger than YPFS in LPS-induced cytokine protein expression. Here, dexamethasone (Dex) served as a positive control, which is a potent synthetic member of the glucocorticoid class of steroid drugs that has anti-inflammatory and immunosuppressant properties. Ten µM Dex could significantly suppress the expressions of pro-inflammatory cytokines up to 20%∼90% in all cases (**Fig. 7B**).

## Discussion

The rational for having specific combination of different herbs in forming a TCM herbal formula is a major stumbling block in the internationalization of Chinese medicine, which hinders the discovery of action mechanisms in using TCM as a therapeutic agent of treating diseases. Here, we disclosed the underlying reasons for the compatibility of different herbs within YPFS by using the analysis of chemical constituents in the extracts. Chemically, YPFS of having 3 different herbs together during the preparation promoted strongly the solubilities of different chemcials, and which was better than single herb or two-herb compositions, i.e. better solubilities of active ingradients in YPFS. Having different herbs together in making a herbal decoction might greatly enhance the pharmaceutical effects by getting more extrable active ingredients in the final decoction. This phenomena of herb compatibility has been revealed similarily in another herbal formula-Danggui Buxue Tang. Having two herbs, Astragali Radix and Angelicae Sinensis Radix, boiling together as Danggui Buxue Tang indeed could enhance the solubilities of the active ingredients [29]. In addition, the decoction showed a robust biological effects in various systems [23], [28]–[30], [32]: these effects were better than the decoction having only single herb. It is worth mentioning that the identification of chemical composition in a herbal decoction would be a pre-requistic step in revealing the possible therapeutic effects. In parallel, the established chemical criteria could serve as parameters for a well-prepared YPFS.

The important role of herb compatibility in YPFS was demonstrated via chemical and biological parameters. Traditionally, the main function of YPFS is enhancing “Qi” that includes “Defensive Qi” referring to the immune function and “Nutritive Qi” referring to nourish blood function. Both of these “Qi” functions could be illustrated here by YPFS. Although we have not illustarted the blood function of YPFS here, this function could be predicted by having AR as well as the bioactive flavonoids. The principal herb of YPFS is AR that contains 0.75∼1.81% by weight of flavonoids including calycosin and formononetin [33]. These two flavonoids are known to induce the gene expression of erythropoietin (EPO), a glycoprotein hormone that controls erythropoiesis of red blood cell production in a dose-dependent manner in cultured fibroblasts [34], as well as in animals (unpublished data). Here, the amount of flavonoids in YPFS was highly enriched to about 4 mg/g of dried YPFS extract: this conentration of flavonoids is expected to perform the blood stimulating function in our body. In parallel, the absorption of flavonoids could be enhanced in cutlured Caco-2 epidermal cells under the influence of AMR extract (unpublished data). These biological activities are all considered as the function of nutritive “Qi”. In defensive “Qi”, the immuno-modulation of YPFS could be in two directions: stimulating and supressing cytokine releases, as shown in this study here.

Although the water extract of SR showed better immune stimulation effect, the role of YPFS in various immune functions could be more balance, i.e. showing effective effects in different aspects of immune responses. The robust immuno-effect of SR extract in either direction could not be beneficency to our body. The functional balance could be a unique character of herbal formula as compared to that of single herbal extract. In order to function effectively, the immune system has to strike a balance between the activities of pro-inflammatory and anti-inflammatory cytokines [35]. Here, we have demonstarted that YPFS possessed both pro- and anti-inflammatory effects in cultured macrophages. The YPFS-induced expressions of pro-inflammatory cytokines are IL-1β, IL-6 and TNFα. In contrast, the YPFS-suppressed expressions of LPS-induced cytokines are IL-1β, IL-6 and TNFα. The herbal decoction exerted bi-directional modulating functions are commonly described previously [36]–[40]; however, this property could be hardly found in the western medicine. In YPFS, the glycoprotein derived from AMR was shown to stimulate markedly the production of TNFα in splenocyte, and in contrast AMR-derived atractylenolide I and atractylenolide III were shown to exert anti-inflammation effects via suppressing the expression of LPS-induced TNFα in cultured macrophages [24], [38]. Although the bi-directional modulating functions of YPFS were demonstrated here, the underlying mechanism was still unknown.

## Materials and Methods

### Chemical and reagent

Astragaloside II, III and IV were purchased from the National Institute for The Control of Pharmaceutical and Biological Products (Beijing, China). Calycosin, calycosin-7-β-D-glucoside, ononin, and formononetin were kindly provided by Prof. Pengfei Tu, Medical College of Peking University. Atractylenolide I, II and III, prim-O-glucosylcimifugin and 5-O-methylvisammioside, scopoletin, isopsoralen and psoralen were purchased from Chendu Biotech Co. (Chendu, China). MTT, LPS, Zymonsan A, dexamethasone and Bay 11-7082 were purchased from Sigma (St. Louis, MO). All these chemicals were over 98% purity. Other culture media and supplements were obtained from Invitrogen Technologies (Carlsbad, CA). Fetal calf serum was from Hyclone (Thermo Fisher Scientific, Walthan MA). MILLIPLEX^®^MAP Kit was obtained from EMD Millipore Corporation (Billerica, MA). IκBα antibody was purchased from Santa Cruz Biotechnology Inc. (Santa Cruz, CA). Glyceraldehyde 3-phosphate dehydrogenase (GAPDH) was obtained from Abcam Ltd. (Cambridge, UK). Enhanced chemiluminesence^TM^ (ECL) was purchased from Amersham Biosciences (Piscataway, NJ). Penicillin, streptomycin, Lipofectamine 2000 and horseradish peroxidase (HRP)-conjugated anti-mouse secondary antibody were purchase from Invitrogen.

### Plant material and preparation of herbal decoction

The roots of *A. membranaceus* var. *mongholicus* (AR), the rhizomes of *A. macrocephala* (AMR) and the roots of *S. divaricata* (SR) were collected from Shanxi province, Anhui province and Heilongjiang province, separately. The authentication of plant materials was performed morphologically by one of the authors, Dr. Tina Dong. Their corresponding voucher specimens, as forms of whole plants, were deposited in Center for Chinese Medicine at Hong Kong University of Science and Technology (HKUST). The raw materials were purchased from medicinal herbal market. No specific permissions were required for the locations or activities during the collection of the raw materials, the location was also not privately-owned or protected. In the preparation of YPFS, the amounts of crude drugs (in slices) of AR, AMR and SR were weighed according to the weight ratio of 1∶2∶1, separately. The herbal mixture was boiled in 8 volumes of water (v/w) by moderate heating for 2 hours. The residues were re-boiled in 6 volumes of water for 1 hour. The extracts pooled from two extractions were filtered, and which were dried by lyophilization and stored at 4°C.

### HPLC method

Fingerprint of YPFS was made by Agilent HPLC system fitted with DAD at 210 nm. The mobile phase consisting of 0.1% phosphate buffer (A) and acetonitrile (B) was eluted at a linear gradient flow of 1 mL/min at 5%–60% B within 60 min. The detection of analytes was accomplished with a C18 column (Inertsil, 4.6×250 mm, 5 µm or equivalent). The herbal extract of YPFS was dissolved in methanol at 40 mg/mL for 30 min sonication, and which was filtered through 0.22 µm filtration membrane before injection.

### LC-MS method

The chemical analysis was performed on an Agilent RRLC 1200 series system (Agilent) equipped with a degasser, a binary pump, an auto-sampler, a DAD and a thermostated column compartment. The herbal extract was separated on an Agilent ZORBAX SB-C18 column (1.8 µm, 4.6×50 mm). An Agilent QQQ-MS/MS (6410A) equipped with an ESI ion source was operated in negative or positive ion mode. The drying gas temperature was 325°C; drying gas flow: 10 L/min; nebulizer pressure: 35 psig; capillary voltage: 4000 V; delta electro multiplier voltage: 400 V. Ten μL (after a 0.45 μm Millipore filter) samples were injected. In a negative mode, a linear gradient elution was applied from 15–20% B at 0–3 min, 20–30% B at 3–8 min, 30–40% B at 8–14 min, 40–75% B at 14–18 min, 15–15% B at 18.5–22 min. In a positive mode, a linear gradient elution was applied from 10–35% B at 0–5.5 min, 35–65% B at 5.5–6 min, 65% B at 6–10 min, 65–80% B at 10–14 min, 10% B at 14.5–18 min. Agilent Mass Hunter workstation software version B.01.00 was used for data acquisition and processing.

### DNA construct and transfection

The pGL4.32 [Luc2P/NF-κB-RE/Hygro] vector (denoted as pNF-κB-Luc) contains five copies of a NF-κB response element (NF-κB-RE) that drives transcription of a luciferase reporter gene luc2P (*Photinus pyralis*) [41]. Luc2P is a synthetically-derived luciferase sequence with humanized codon optimization that is designed for high expression and reduced anomalous transcription. The Luc2P gene contains hPEST, a protein destabilization sequence. The protein encoded by luc2P responds more quickly than the protein encoded by Luc2 gene upon induction. The vector backbone contains an ampicillin resistance gene to allow selection in *E. coli* and a mammalian selectable marker for hygromycin resistance.

RAW 264.7 murine macrophages (American Type Culture Collection, Manassas, VA) were cultured in high-glucose Dulbecco's Modified Eagle's medium supplemented with 100 U/mL penicillin/streptomycin, 10% heat in-active fetal bovine serum (all from Invitrogen). The density of Raw 264.7 cells in 24-well plate was 5,000/well. Cells were incubated under 5% CO_2_ at 37°C for 24 hours before the transfection. pNF-κB-Luc was transfected to cultured RAW 264.7 macrophages by Lipofectamine 2000. Twently-four hours later, the transfected cells were challenged with different herbal extracts. The transfection efficient was over 80%, as determined by another control plasmid of having a β-galactosidase, under a cytomegalovirus enhancer promoter.

### Quantitative real-time PCR

For the analyses of IL-1β, IL-6 and TNFα mRNA expressions in cultured macrophages, the cultures were treated with the herbal extracts. Total RNA was isolated by TRIzol reagent and reverse transcribed into cDNAs according to the manufacturer's instructions (Invitrogen). Real-time PCR was performed by using SYBR Green Master mix and ROX reference dye according to the manufacturer's instructions (Applied Bioscience, Foster city, CA). The primers were: 5′-AAA TAC CTG TGG CCT TG-3′ and 5′-TTA GGA AGA CAC GGA TTC-3′ for murine IL-1β (296 bp; NM_008361); 5′-GGA GTA CCA TAG CTA CCT GG-3′ and 5′-CTA GGT TTG CCG AGT AGA TC-3′ for murine IL-6 (283 bp; NM_031168); 5′- AGT GAC AAG CCT GTA GCC -3′ and 5′- AGG TTG ACT TTC TCC TGG-3′ for murine TNFα (251 bp; NM_013693); The GAPDH was used as an internal control in all cases, and its primer sequences were 5′- AAC GGA TTT GGC CGT ATT GG-3′ and 5′- CTT CCC GTT CAG CTC TGG G-3′ (657 bp; NR_0215885). SYBR green signal was detected by M×3000ptm multiplex quantitative PCR machine (Stratagene, La Jolla, CA). Transcript levels were quantified, where the values of target genes were normalized by GAPDH expression in the same sample at first before comparison. The PCR products were analyzed by gel electrophoresis and melting curve analysis to confirm the specific amplification.

### Luciferase activity assay

After transfection with the luciferase reporter gene construct and drug treatment for 24 hours, the cells were stored frozen in −80°C, or solubilized in lysis buffer (100 mM potassium phosphate buffer, pH 7.8, containing 1 mM DTT) immediately and transferred into 1.5-mL eppendorf tubes. After vortex for 10 min in 4°C, the lysates were centrifuged in 13,200×g for 5 min in 4°C. Sixty μL of the cell lysates was transferred to the assay plate and set on the luminance reading machine (FLUOStar OPTIMA, Germany), which would automatically add luciferase reagent A and B (Invitrogen) into the lysates, and activated the luciferase activity. The readings of luminance intensity were equalized by the protein concentration of lysates, and the data indicated to the luciferase activities of the samples.

### Cytokine profile assay

In determining the expressions of pro-inflammatory cytokines, 50,000 macrophages were cultured for 48 hours with the herbal extracts. For the study on anti-inflammation effects of YPFS, 80,000 macrophages were LPS-primed for 24 hours followed by pre-treatment with the herbal extracts for 3 hours. Supernatants of culture medium were collected to measure the concentrations of pro-inflammatory cytokines (IL-1β, IL-6 and TNFα) using Milliplex® technology. The Milliplex mouse cytokine kit pre-mixed 3-plex (Millipore, MA) was used to determine the cytokine levels in cultured medium according to the manufacturer's instructions.

### Western blot analysis

Macrophages were treated with drugs at different time (0, 10, 20 and 30 min), and then cultures were collected immediately in direct lysis buffer (0.125 M Tris-Cl, pH 6.8, 4% SDS, 20% glycerol, 2% 2-mercaptoethanol and 0.02% bromophenol blue) and heated at 100°C for 10 min. Cellular protein from treated and untreated cell extracts was electro-blotted onto a nitrocellulose membrane following separation on a 10% SDS-PAGE. The immune-blot was incubated with blocking solution (5% BSA) for 2 hours at room temperature, followed by incubation for overnight with a primary antibody (1: 1000 for IκBα, 1: 50,000,00 for GAPDH) at 4°C. After intensive washing with Tween 20/Tris-buffered saline (TBS-T) for 4 times, a 1∶5,000 dilution of HRP-conjugated anti-mouse antibody was added and incubated for 1.5 hours at room temperature. Blots were washed 4 times with TBS-T, and then developed by ECL method. The intensities of the bands in the control and different samples, run on the same gel and under strictly standardized ECL conditions, were compared on an image analyzer, using a calibration plot constructed from a parallel gel with serial dilutions of one of the samples.

### Phagocytosis assay

The phagocytic capability of cultured macrophages was measured by using the Vybrant Phagocytosis Assay Kit (Molecular Probes, Eugene, OR). Macrophages (10^5^/well) were incubated with fluorescein-labeled *E. coli* K-12 bio-particles and intracellular uptake was quantified by measuring fluorescence emitted by engulfed particles. Extracellular fluorescence was quenched by trypan blue. The trypan blue was then removed, and the amount of bio-particles engulfed by the cells was quantitatively measured using Spectra Max Gemini EM ELISA plate reader at 480 nm excitation and 520 nm emission.

### Statistical analysis and other assays

The protein concentrations were measured routinely by Bradford's method (Hercules, CA). Statistical analyses were performed using one way ANOVA followed by the student t-test. Statistically significant changes were classed as [*] where p<0.05; [**] where p<0.01 and; [***] where p<0.001.

## Supporting Information

Figure S1 **Chemical structures of markers analyzed in YPFS.** (**A**): The chemical structures of compounds derived from AR were shown; (**B**): The chemical structures of compounds derived from AMR were shown; (**C**): The chemical structures of compounds derived from SR were shown; (**D**): Internal standard for determination of markers analyzed in the negative mode, including aesculetin for scopoletin, gingenoside Rg1 for astragaloside II, III and IV, and chrysin for calycosin, calycosin-glucoside, ononin and formononetin; Internal standard for determination of markers analyzed in the positive mode, including esculin for prim-O-glucosylcimifugin, 5-O-methylvisammioside, psoralen and isopsoralen, while cryptotanshinone for atractylenolide I, II and III. The numbers were referring to the LC analysis in **Figs. 1** and **2**.
(TIF)

Figure S2 **The cytotoxicity of herbal extracts on cultured macrophages.** Cultured macrophages were seeded on to 96-well plate and incubated for 24 hours. After that, the cells were treated with herbal extracts in different concentrations (0.03 mg/mL-3 mg/mL) for 24 hours. The MTT solution was added to the cell cultures and incubated for 1 hour at 37°C. Absorbance was measured at 570 nm in a microplate reader. Values are expressed as the % of total cell number against the control, and in Mean ± SD, *n* = 3.
(TIF)

Table S1 **Mass spectra properties of chemical markers in YPFS in negative mode.** (**A**): The detected chemicals had the greatest responses under the negative mode: the [M−H]^−^ was used as the precursor ion; (**B**): The fragmentor energy was optimized to have the greatest ionize efficiency; (**C**): The collision energy was optimized to have the greatest product ion intensity, which was the key factor in the MRM mode; (**D**): Two product ions were used for the MRM analysis. The upper one was used for quantitative analysis and the lower one was for qualitative analysis, which could guarantee the precision of analytes; (**E**): The retention time was determined by 3 different individual analyses (*n* = 3); (**F**): The precursor ion of chemical marker was [M+Cl−H]^−^ under the negative mode. (**G**): The precursor ion of astragaloside IV was [M+HCOOH−H]^−^ under the negative mode.
(DOC)

Table S2 **Mass spectra properties of chemical markers in YPFS in positive mode.** (**A**): The detected chemicals had the greatest responses under the positive mode: the [M+H]^+^ was used as the precursor ion; (**B**): The fragmentor energy was optimized to have the greatest ionize efficiency; (**C**): The collision energy was optimized to have the greatest product ion intensity, which was the key factor in the MRM mode; (**D**): Two product ions were used for the MRM analysis. The upper one was used for quantitative analysis and the lower one was for qualitative analysis, which could guarantee the precision of analytes; (**E**): The retention time was determined by 3 different individual analyses (*n* = 3).
(DOC)

Table S3 **Calibration curves, LOD and LOQ for fifteen chemicals in YPFS.** (**A**): These calibration curves were constructed by plotting the peak area versus the concentration of each analyte. Each calibration curve was derived from six data points, *n* = 3, and the SD was <5% of the Mean; (**B**): LOD refers to the limits of detection; (**C**): LOQ refers to the limits of quantification.
(DOC)

Table S4 **Precision, repeatability and recovery of fifteen markers in YPFS.** (**A**): Recovery (%)  = 100 X (amount found−original amount)/amount spiked. The data was presented as average of three independent determinations, and the SD was <5% of the Mean, which was not shown for clarity; (**B**): The intra-day analysis refers to the sample examined for six replicates within one day; (**C**): The inter-day analysis refers to the sample examined in triplicates over three consecutive days.
(DOC)
