# Supplementary figures and images for: Yu Ping Feng San, an Ancient Chinese Herbal Decoction Containing Astragali Radix, Atractylodis Macrocephalae Rhizoma and Saposhnikoviae Radix, Regulates the Release of Cytokines in Murine Macrophages
Source: PLoS One. 2013 Nov 11;8(11):e78622. doi: 10.1371/journal.pone.0078622 (PMC3823765; doi:10.1371/journal.pone.0078622)

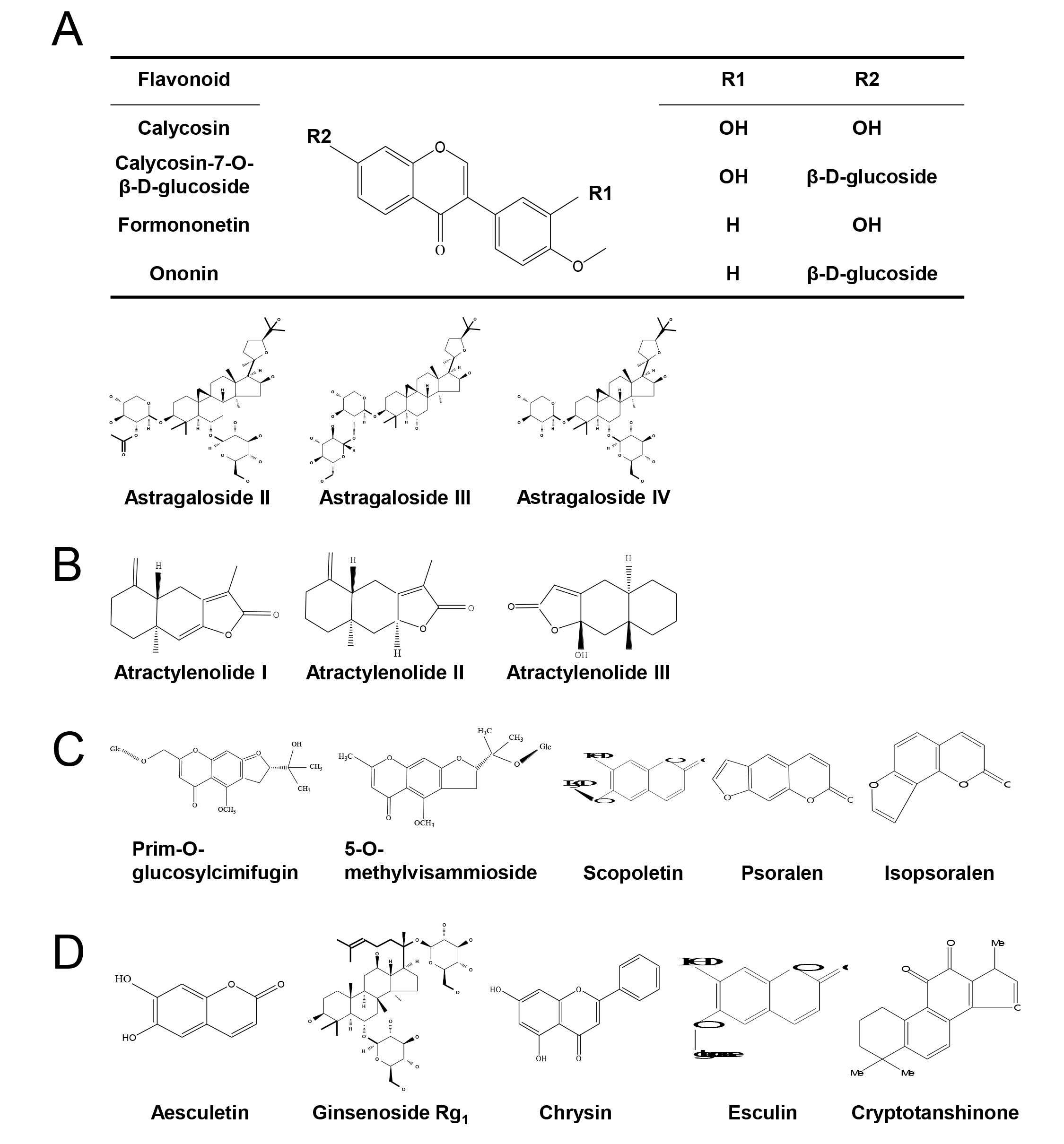

Supplement: Figure S1 — Chemical structures of markers analyzed in YPFS. (A): The chemical structures of compounds derived from AR were shown; (B): The chemical structures of compounds derived from AMR were shown; (C): The chemical structures of compounds derived from SR were shown; (D): Internal standard for determination of markers analyzed in the negative mode, including aesculetin for scopoletin, gingenoside Rg1 for astragaloside II, III and IV, and chrysin for calycosin, calycosin-glucoside, ononin and formononetin; Internal standard for determination of markers analyzed in the positive mode, including esculin for prim-O-glucosylcimifugin, 5-O-methylvisammioside, psoralen and isopsoralen, while cryptotanshinone for atractylenolide I, II and III. The numbers were referring to the LC analysis in Figs. 1 and 2 . (TIF) [file pone.0078622.s001.tif]

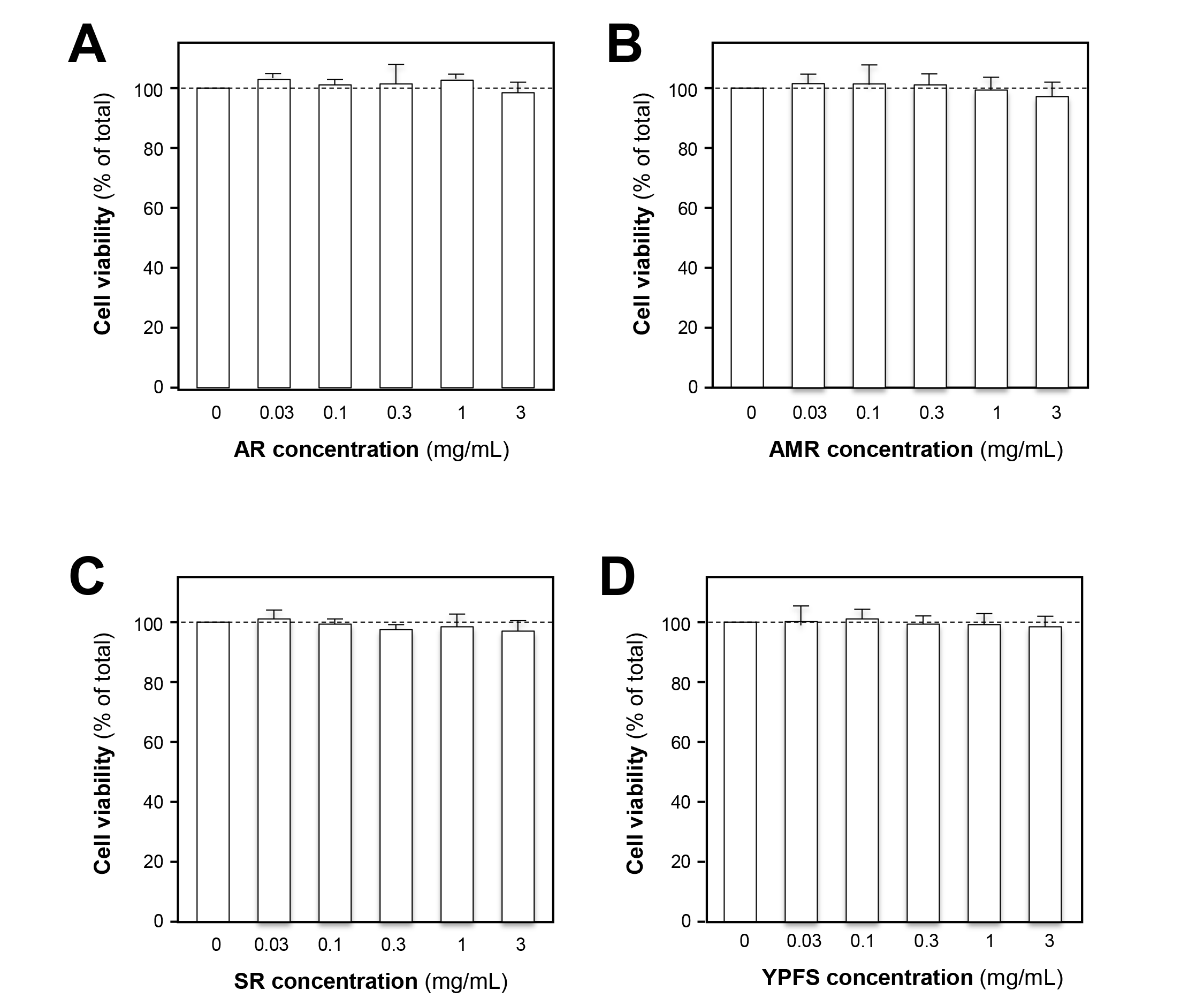

Supplement: Figure S2 — The cytotoxicity of herbal extracts on cultured macrophages. Cultured macrophages were seeded on to 96-well plate and incubated for 24 hours. After that, the cells were treated with herbal extracts in different concentrations (0.03 mg/mL-3 mg/mL) for 24 hours. The MTT solution was added to the cell cultures and incubated for 1 hour at 37°C. Absorbance was measured at 570 nm in a microplate reader. Values are expressed as the % of total cell number against the control, and in Mean ± SD, n = 3. (TIF) [file pone.0078622.s002.tif]
