# Supplementary material for: Yu Ping Feng San, an Ancient Chinese Herbal Decoction Containing Astragali Radix, Atractylodis Macrocephalae Rhizoma and Saposhnikoviae Radix, Regulates the Release of Cytokines in Murine Macrophages
Source: PLoS One. 2013 Nov 11;8(11):e78622. doi: 10.1371/journal.pone.0078622 (PMC3823765; doi:10.1371/journal.pone.0078622)
Supplement: Table S1 — Mass spectra properties of chemical markers in YPFS in negative mode. (A): The detected chemicals had the greatest responses under the negative mode: the [M−H]− was used as the precursor ion; (B): The fragmentor energy was optimized to have the greatest ionize efficiency; (C): The collision energy was optimized to have the greatest product ion intensity, which was the key factor in the MRM mode; (D): Two product ions were used for the MRM analysis. The upper one was used for quantitative analysis and the lower one was for qualitative analysis, which could guarantee the precision of analytes; (E): The retention time was determined by 3 different individual analyses (n = 3); (F): The precursor ion of chemical marker was [M+Cl−H]− under the negative mode. (G): The precursor ion of astragaloside IV was [M+HCOOH−H]− under the negative mode. (DOC) [file pone.0078622.s003.doc]

**Table S1 Mass spectra properties of chemical markers in YPFS in negative mode.**

| **Chemical** | **Formula** | **Calculated mass[M]** | **Precursor ion[M-H]^A^** | **Fragmentor Energy^B^** | **Collison energy^C^** | **Product**  **ion^D^** | **Retention time(min)^E^** |
| --- | --- | --- | --- | --- | --- | --- | --- |
| **Calycosin-7-O-β-D-glucoside^F^** | C_22_H_22_O_10_ | 446.40 | 481.00 | 120 | 5 27 | 283.1 268.1 | 4.663 |
| **Scopoletin** | C_10_H_8_O_4_ | 192.04 | 191.03 | 106 | 9 21 | 176.0 104.0 | 5.866 |
| **Ononin^F^** | C_22_H_22_O_9_ | 430.40 | 465.10 | 80 | 5 23 | 267.1 252.1 | 8.090 |
| **Calycosin** | C_16_H_12_O_5_ | 284.10 | 283.10 | 100 | 13 29 | 268.9 211.6 | 10.134 |
| **Astragaloside IV^G^** | C_41_H_68_O_14_ | 784.90 | 829.50 | 190 | 5 25 | 829.5 783.2 | 12.653 |
| **Astragaloside III** | C_41_H_68_O_14_ | 784.93 | 819.40 | 230 | 5 25 | 819.4 783.4 | 12. 362 |
| **Astragaloside II** | C_43_H_70_O_15_ | 827.02 | 861.20 | 170 | 5 | 861.2 | 13.752 |
| **Formononetin** | C_16_H_12_O_4_ | 268.10 | 267.10 | 150 | 17 29 | 252.8 223.9 | 14.376 |
| **Aesculetin** | C_9_H_6_O_4 IS_ | 178.14 | 177.00 | 106 | 13 17 | 133.0 105.0 | 3.514 |
| **Ginsenoside Rg_1_** | C_42_H_72_O_24 IS_ | 800.50 | 799.50 | 250 | 5 21 | 799.5 637.3 | 7.446 |
| **Chrysin** | C_15_H_10_O_4 IS_ | 254.07 | 253.06 | 106 | 29 57 | 143.0 63.0 | 16.666 |
