# Supplementary material for: Yu Ping Feng San, an Ancient Chinese Herbal Decoction Containing Astragali Radix, Atractylodis Macrocephalae Rhizoma and Saposhnikoviae Radix, Regulates the Release of Cytokines in Murine Macrophages
Source: PLoS One. 2013 Nov 11;8(11):e78622. doi: 10.1371/journal.pone.0078622 (PMC3823765; doi:10.1371/journal.pone.0078622)
Supplement: Table S2 — Mass spectra properties of chemical markers in YPFS in positive mode. (A): The detected chemicals had the greatest responses under the positive mode: the [M+H]+ was used as the precursor ion; (B): The fragmentor energy was optimized to have the greatest ionize efficiency; (C): The collision energy was optimized to have the greatest product ion intensity, which was the key factor in the MRM mode; (D): Two product ions were used for the MRM analysis. The upper one was used for quantitative analysis and the lower one was for qualitative analysis, which could guarantee the precision of analytes; (E): The retention time was determined by 3 different individual analyses (n = 3). (DOC) [file pone.0078622.s004.doc]

| **Chemical** | **Formula** | **Calculatedmass[M]** | **Precursor ion[M+H]^A^** | **FragmentorEnergy^B^** | **Collison energy^C^** | **Product ion^D^** | **Retention time(min)^E^** |
| --- | --- | --- | --- | --- | --- | --- | --- |
| **Prim-O-glucosyl-cimifugin** | C_22_H_28_O_11_ | 468.45 | 469.20 | 202 | 29 57 | 307.0 259.0 | 4.051 |
| **5-O-methlyvisa-mmioside** | C_22_H_28_O_10_ | 452.45 | 453.20 | 154 | 25 25 | 291.1 273.1 | 5.025 |
| **Psoralen** | C_11_H_6_O_3_ | 186.03 | 187.04 | 154 | 25 21 | 131.1 115.1 | 7.752 |
| **Isopsoralen** | C_11_H_6_O_3_ | 186.03 | 187.04 | 154 | 25 45 | 131.1 77.0 | 7.756 |
| **Atractylenolide III** | C_15_H_20_O_3_ | 248.14 | 249.15 | 58 | 5 65 | 231.1 77.0 | 8.947 |
| **Atractylenolide II** | C_15_H_20_O_2_ | 232.15 | 233.16 | 106 | 29 41 | 105.0 91.0 | 11.186 |
| **Atractylenolide I** | C_15_H_18_O_2_ | 230.13 | 231.14 | 200 | 5 57 | 128.0 115.1 | 10.992 |
| **Esculin** | C_15_H_16_O_9 IS_ | 340.29 | 341.10 | 106 | 13 49 | 179.0 123.0 | 2.883 |
| **Crytotanshinone** | C_19_H_20_O_3 IS_ | 296.36 | 297.20 | 106 | 21 21 | 254.0 251.1 | 12.673 |

**Table S2 Mass spectra properties of chemical markers in YPFS in positive mode.**
