# Supplementary material for: Yu Ping Feng San, an Ancient Chinese Herbal Decoction Containing Astragali Radix, Atractylodis Macrocephalae Rhizoma and Saposhnikoviae Radix, Regulates the Release of Cytokines in Murine Macrophages
Source: PLoS One. 2013 Nov 11;8(11):e78622. doi: 10.1371/journal.pone.0078622 (PMC3823765; doi:10.1371/journal.pone.0078622)
Supplement: Table S3 — Calibration curves, LOD and LOQ for fifteen chemicals in YPFS. (A): These calibration curves were constructed by plotting the peak area versus the concentration of each analyte. Each calibration curve was derived from six data points, n = 3, and the SD was <5% of the Mean; (B): LOD refers to the limits of detection; (C): LOQ refers to the limits of quantification. (DOC) [file pone.0078622.s005.doc]

**Table S3 Calibration curves, LOD and LOQ for fifteen chemicals in YPFS.**

| **Chemical** | **Calibration curve ^A^** | **Correlation coefficient (*r*^2^)** | **Linear range (ng/mL)** | **LOD ^B^ (ng/mL)** | **LOQ ^C^ (ng/mL)** |
| --- | --- | --- | --- | --- | --- |
| **Calycosin** | y = 2211.3x + 16987.2 | 0.9995 | 2-2000 | 0.36 | 0.83 |
| **Calycosin-7-O-β-D-glucoside** | y = 297.7x + 333.3 | 0.9997 | 1-1000 | 0.99 | 1.78 |
| **Formononetin** | y = 362.5x – 3304.2 | 0.9993 | 2-2000 | 0.41 | 0.75 |
| **Ononin** | y = 428.3x + 1407.5 | 0.9975 | 1-1000 | 1.04 | 2.09 |
| **Astragaloside II** | y = 101.3x + 335.6 | 0.9978 | 10-1000 | 3.58 | 8.97 |
| **Astragaloside III** | y = 13.87x + 15.86 | 0.9930 | 1-1000 | 0.55 | 2.04 |
| **Astragaloside IV** | y = 1624.6x - 5385.8 | 0.9999 | 1-1000 | 0.66 | 1.19 |
| **Atractylenolide I** | y = 65.2x + 37.9 | 0.9995 | 1-2000 | 0.86 | 2.38 |
| **Atractylenolide II** | y = 432.9x + 5287.1 | 0.9987 | 2-2000 | 0.83 | 1.88 |
| **Atractylenolide III** | y = 756.8x - 11150.7 | 0.9996 | 2-2000 | 0.72 | 1.04 |
| **Prim-O-glucosylcimifugin** | y = 1472.2x + 7244.5 | 0.9996 | 2-2000 | 0.75 | 1.02 |
| **5-O-methylvisammioside** | y = 2944.9x + 41840.5 | 0.9985 | 2-2000 | 0.59 | 0.98 |
| **Scopoletin** | y = 588.1x + 3437.9 | 0.9999 | 1-2000 | 0.37 | 0.99 |
| **Psoralen** | y = 1021.9x – 1280.9 | 0.9999 | 1-1000 | 0.52 | 1.14 |
| **Isopsoralen** | y = 4889.6x - 3082.7 | 0.9998 | 1-1000 | 0.35 | 1.03 |
