# Supplementary material for: Yu Ping Feng San, an Ancient Chinese Herbal Decoction Containing Astragali Radix, Atractylodis Macrocephalae Rhizoma and Saposhnikoviae Radix, Regulates the Release of Cytokines in Murine Macrophages
Source: PLoS One. 2013 Nov 11;8(11):e78622. doi: 10.1371/journal.pone.0078622 (PMC3823765; doi:10.1371/journal.pone.0078622)
Supplement: Table S4 — Precision, repeatability and recovery of fifteen markers in YPFS. (A): Recovery (%) = 100 X (amount found−original amount)/amount spiked. The data was presented as average of three independent determinations, and the SD was <5% of the Mean, which was not shown for clarity; (B): The intra-day analysis refers to the sample examined for six replicates within one day; (C): The inter-day analysis refers to the sample examined in triplicates over three consecutive days. (DOC) [file pone.0078622.s006.doc]

**Table S4 Precision, repeatability and recovery of fifteen markers in YPFS.**

|  | **Precision** | | | | **Repeatability (*n* = 6)** | | **Recovery^A^ (*n*=3)** | |
| --- | --- | --- | --- | --- | --- | --- | --- | --- |
| **Chemical** | **Intra-day (*n*=6)^B^** | | **Inter-day (*n*=6)^C^** | |  |  |  |  |
|  | **Mean (ng/mL)** | **RSD (%)** | **Mean (ng/mL)** | **RSD (%)** | **Mean (ng/mL)** | **RSD (%)** | **Mean (ng/mL)** | **RSD (%)** |
| **Calycosin** | 100.53 | 1.05 | 99.85 | 1.95 | 407.43 | 1.22 | 96.91 | 2.26 |
| **Calycosin-7-O-β-D-glucoside** | 99.89 | 1.73 | 100.56 | 1.12 | 1889.65 | 3.87 | 102.78 | 3.32 |
| **Formononetin** | 100.57 | 1.17 | 99.39 | 1.67 | 1139.69 | 0.79 | 96.78 | 1.78 |
| **Ononin** | 100.03 | 1.57 | 99.69 | 2.51 | 550.89 | 1.65 | 97.36 | 2.99 |
| **Astragaloside II** | 101.03 | 2.99 | 99.05 | 3.76 | 2899.13 | 3.90 | 103.71 | 3.12 |
| **Astragaloside III** | 100.65 | 2.03 | 99.14 | 2.47 | 453.73 | 2.83 | 98.98 | 2.90 |
| **Astragaloside IV** | 100.12 | 1.28 | 100.76 | 2.47 | 380.83 | 2.45 | 97.11 | 3.13 |
| **Atractylenolide I** | 99.95 | 1.78 | 100.13 | 1.92 | 86.47 | 2.26 | 97.49 | 1.02 |
| **Atractylenolide II** | 100.09 | 1.21 | 99.53 | 1.49 | 107.07 | 1.32 | 102.56 | 2.37 |
| **Atractylenolide III** | 99.79 | 0.74 | 100.16 | 2.25 | 760.66 | 1.72 | 100.81 | 1.35 |
| **Prim-O-glucosylcimifugin** | 99.09 | 0.53 | 99.89 | 2.11 | 2097.74 | 1.43 | 100.42 | 1.23 |
| **5-O-methylvisammioside** | 100.34 | 0.65 | 100.14 | 2.00 | 1462.92 | 1.63 | 100.55 | 1.71 |
| **Scopoletin** | 99.91 | 0.32 | 100.17 | 1.89 | 17.83 | 1.59 | 100.09 | 1.88 |
| **Psoralen** | 100.08 | 0.81 | 99.97 | 1.10 | 25.21 | 2.84 | 98.03 | 3.43 |
| **Isopsoralen** | 100.12 | 0.69 | 100.10 | 2.35 | 26.01 | 2.19 | 97.36 | 3.44 |
